# The cost of risk score and star rating growth: insights for future Medicare advantage innovation models

**DOI:** 10.1093/haschl/qxag199

**Published:** 2026-07-31

**Authors:** Brett Lissenden, Allison Dorneo, John Robst, Peter F Lyu

**Affiliations:** RTI International, Research Triangle Park, NC 27709, United States; RTI International, Research Triangle Park, NC 27709, United States; RTI International, Research Triangle Park, NC 27709, United States; RTI International, Research Triangle Park, NC 27709, United States

**Keywords:** Medicare advantage (MA), value-based insurance design (VBID), risk scores, star ratings

## Abstract

**Introduction:**

The Centers for Medicare and Medicaid Services (CMS) may test a new Medicare Advantage (MA) innovation model for achieving broader Medicare efficiency. Informed by the MA value-based insurance design (VBID) model experience, we simulated minimum bid discounts and discussed strategies that would be needed to offset potential costs from plan risk score and star rating growth effects.

**Methods:**

Using VBID evaluation data, we quantified the challenge CMS faces related to risk score and star ratings growth by calculating simulated payments to plans and bid discounts needed to achieve target savings.

**Results:**

Had CMS imposed minimum bid discounts (holding model participation constant), we estimated that an average 7% bid discount for VBID plans could have achieved budget neutrality. Alternatively, to achieve 2% CMS savings, VBID would have required 14.5% lower plan bids.

**Conclusion:**

Small risk score and star rating growth can jeopardize the sustainability and value of MA innovation models, and only a small share of bid discounts pass through to Medicare under MA payment policies. To achieve savings in future MA innovation models, we propose a combination of design features like minimum bid discounts, alternate risk score and star rating methodologies, stopgap measures, and budget neutrality constraints.

Key Points

- Under the VBID model, relatively small increases in MA risk scores and modest gains in star ratings combined with minimal plan bid reductions resulted in significant costs to the Medicare program.
- VBID would have needed average bid discounts of 7% to offset costs driven by 1.5% and 5.1% growth in risk scores and star ratings, respectively.
- Future MA innovation models may test direct payment methodology reforms (eg, minimum bid discounts, alternative approaches to risk adjustment and quality bonuses that are more budget neutral) or include model stopgaps (eg, corridors or fixed baselines) to ensure plan payments align with model goals and objectives.

## Introduction

With most Medicare beneficiaries enrolled in Medicare Advantage (MA), MA is well-positioned for innovations that can improve the cost and quality of care for enrollees. To date, the Centers for Medicare and Medicaid Services (CMS) has only tested one MA-focused innovation, the value-based insurance design (VBID) model. VBID was first implemented in 2017 across 9 insurers and 45 health plans.^1^ VBID allowed participating plans to offer enhanced supplemental benefits, reduced cost sharing, and incentives for engaging in health-related activities and services, with the goals of promoting high-value care and avoiding preventable, high-cost events.^1-3^ In short, CMS aimed to generate value from VBID through a combination of quality improvements and bid reductions from participating plans via expanded benefit flexibilities.

By 2023, nearly 25% of MA plans nationwide participated in VBID (1218 plans among 46 insurers).^1^ In spite of robust model participation, VBID evaluations found that the model cost CMS over $4 billion between 2021 and 2022.^4^ CMS terminated VBID at the end of 2025 due to the “substantial and unmitigable costs to the Medicare Trust Funds.”^3,4^

Though VBID demonstrated promise for improving the quality of care, differential increases in participating plans' risk scores and star ratings amplified existing problems facing the broader MA program, thereby compromising VBID sustainability.^4,5^ In 2022, VBID was associated with an average 1.5% increase in beneficiaries risk scores and an average 5.1% increase in overall star ratings, relative to a comparison group of non-VBID plans.^1^ Under the current MA program, higher risk scores and higher star ratings result in higher payments to plans.

Although growth in risk scores and star ratings might have been related to improvements in care like medication adherence and chronic care management, they also led to meaningful costs to Medicare, and VBID's payment design failed to prevent or recoup those costs.^1-3^ In 2022, evaluators found that VBID plans had only marginally reduced their standardized bids (the basis of plan payments and estimates of cost of care to MA organizations) by 0.6% relative to non-VBID plans.^1^ These cost-of-care discounts were ultimately insufficient to offset increased CMS spending driven by higher risk scores and star ratings, highlighting the model's limited net value and the challenges of achieving cost neutrality in future innovation models.

While the VBID evaluation reports quantify what the impact of the model was, we quantified what impact would have been needed for financial sustainability. Using similar parameters as the VBID model evaluation, we calculated the minimum bid discounts required to maintain budget neutrality or achieve target savings for CMS given varying growth in average risk scores and star ratings. Using these results, we consider the lessons learned from VBID and the potential model design features that CMS can apply in future innovation models.

## Data and methods

### Data

We collected public data on plan payments and findings from the VBID evaluation report, the star ratings fact sheet, and CMS rate announcement for 2022.^1,6,7^ To simulate total CMS payments to the average VBID plan, we identified key national average parameters from our data: an average per-member-per-month (PMPM) benchmark ($1028.38 PMPM), coding intensity adjustment (0.94), base standardized PMPM bid ($905 PMPM) (for non-VBID plans), base average CMS-HCC risk score (1.278), and base distribution of plans across star rating scores (eg, 31% of beneficiaries in 4-star plans). We varied three parameters reflecting effects from VBID participation: percent growth in average risk score from the base, percent growth in average star rating from the base (see Technical Appendix), and percent PMPM dollar change in standardized bid from the base.

### Simulation methodology

We simulated the average losses/savings of a hypothetical VBID model under different scenarios defined by a continuous range of positive values for risk score growth effect (ie, growth due to VBID participation above what would be expected absent participation; 0% to 1.5%) and star rating growth effect (0% to 5.1%). VBID's growth estimates were treated as upper bounds since our focus was on policies to mitigate growth; we considered higher growth factors in the Technical Appendix. We defined CMS losses/savings as the incrementally higher/lower PMPM plan payments under a particular simulation relative to the base case. We then grid-searched a continuous range of percent decreases in standardized bid (0% to 20%) to identify the minimum discounts that would, respectively, achieve two different target outcomes: budget neutrality (0% savings/losses) and 2% savings. We followed established formulas to calculate plans' base payments, rebates, and total payments across these parameters (see Technical Appendix). Across all combinations of risk score and star rating growth, we reported the required standardized plan discounts via heat maps.

There are several limitations to our analysis using national average parameters specific to the VBID model. First, future MA innovation models may elicit risk score and star rating growth differently than VBID. For example, the minimum bid discounts and other design features we discuss would likely impact plan participation, which we did not explicitly model in our analysis. In a voluntary setting, optimal minimum bid discounts and policy levers would need to balance CMS savings with participation incentives. Second, our simulations mask plan-level heterogeneity that may be associated with participation and influence the relationship between risk score and star rating growth and required bid discounts. To address these concerns, we conducted several sensitivity analyses adjusting our base parameters (eg, average standardized bid amounts, star rating distribution) and growth factors to test how results vary under different scenarios.

## Results

**Figure 1:**
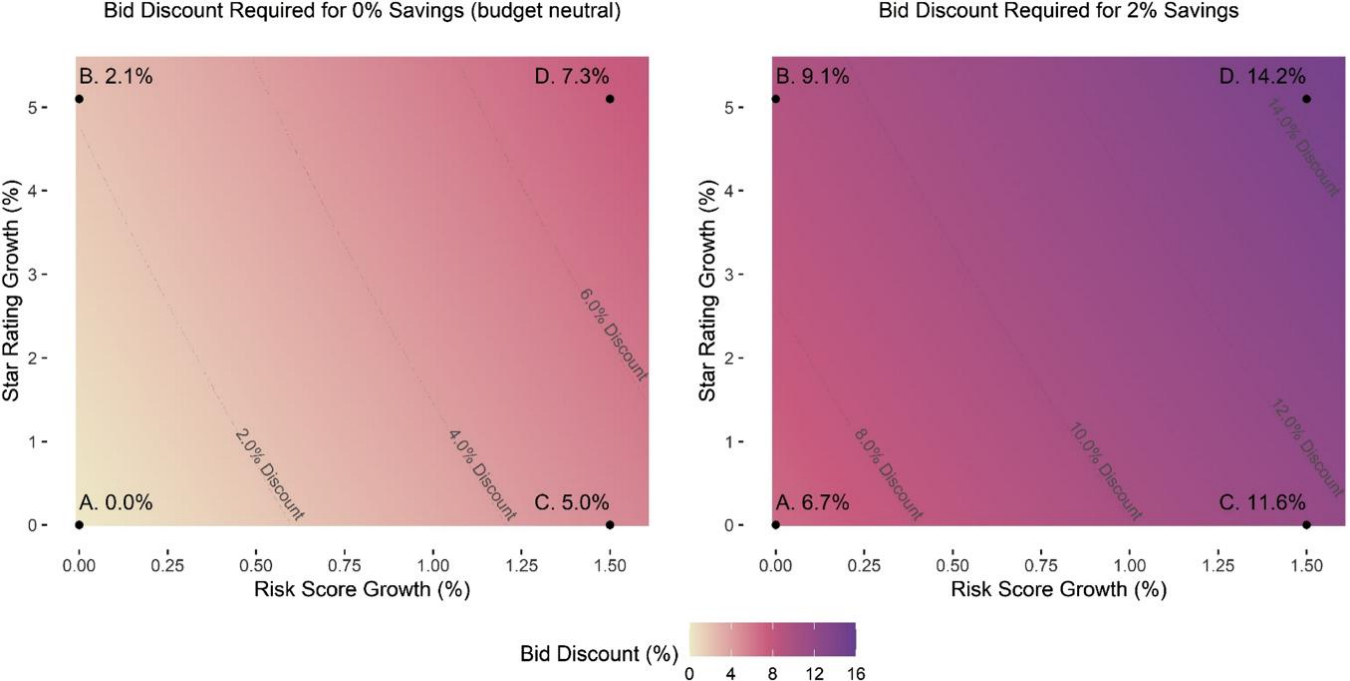
Standardized plan bid discounts needed for CMS budget neutrality and target savings, with varying risk score and star ratings growth. Notes: VBID = value-based insurance design, CMS = Centers for Medicare and Medicaid Services. The dotted lines reflect the % bid discounts needed to achieve either 0% or 2% PMPM savings corresponding to varying degrees of risk score and star rating growth. Darker shaded regions reflect areas in which greater bid discounts are needed to achieve savings. The standardized bid amount for non-VBID plans was an estimated $905 PMPM in 2022.

We illustrate our simulation results in Figure 1; Panel 1 illustrates the bid discounts required to achieve budget neutrality, and Panel 2 illustrates discounts required to achieve 2% savings. In Figure 1, the horizontal axes reflect risk score growth effect, and the vertical axes reflect star rating growth effect. The shade of color represents the minimum standardized bid discount that achieved target CMS savings (0% in Panel 1 or 2% in Panel 2) for a given combination of risk score and star rating growth. Darker colors indicate higher standardized bid discounts. The dashed lines connect the scenarios with equal minimum discount amounts, ranging from 2% to 6% in Panel 1% and 8% to 14% in Panel 2.

We also highlight four specific scenarios: Scenario A reflects zero growth in risk scores and star ratings, Scenario B reflects no risk score growth but 5.1% growth in average star rating, Scenario C reflects no star rating growth but 1.5% growth in average risk scores, and Scenario D reflects 1.5% growth in average risk scores and 5.1% growth in average star ratings, mimicking the original VBID model and assuming associated growth observed in the VBID evaluation were causal effects.

With zero risk score or star rating growth, in both panels, any standardized bid discount would yield positive CMS savings, but achieving 2% savings required a 6.7% bid discount (Panel 2, Scenario A). Assuming 5.1% star rating growth, as estimated in the VBID evaluation, and no risk score growth, a 2.1% standardized bid discount was needed for budget-neutrality (Panel 1, Scenario B) and a 9.1% discount needed for 2% savings (Panel 2, Scenario B). With no star rating growth but 1.5% risk score growth, simulated plans had to offer a 5.0% standardized bid discount to allow budget-neutrality (Panel 1, Scenario C) and an 11.6% discount for 2% savings (Panel 2, Scenario C). With both 5.1% star rating growth and 1.5% risk score growth, our simulation required a 7.3% standardized bid discount to achieve budget-neutrality (Panel 1, Scenario D) and a 14.2% discount for 2% savings (Panel 2, Scenario D).

Sensitivity analyses adjusting our base parameters (eg, standardized bid amounts, star ratings distribution) and growth factors indicated that the relationship between risk score and star rating growth and required discounts is robust and largely generalizable across varying plan characteristics and levels of growth (Technical Appendix, Table A2, Figure A1).

## Discussion

As the CMS Innovation Center (CMMI) develops new MA innovation models to improve the MA program, they will need to consider payment design features that help plans achieve model goals, safeguard CMS spending, and ensure model sustainability in the face of higher costs related to higher risk scores and star ratings. We recommend that CMMI consider a combination of design features.

First, CMS could impose a minimum bid discount for participating plans. Other, non-MA, CMMI models include minimum discount factors, for example, ranging from 1.5% to 2% in the Transforming Episode Accountability Model (TEAM).^8^ A similar minimum bid discount could have improved CMS financial outcomes with VBID but, according to our simulations, may not have been sufficient for CMS budget neutrality. Under VBID parameters, a 2% bid discount would have required less than 0.6% risk score growth for CMS budget neutrality. In determining the minimum threshold, CMS would need to balance fiscal protection with participation disincentives, as setting minimum discounts too high will diminish incentives for plans to join a voluntary model.

Second, CMS could restructure rebate percentages to increase CMS' average savings capture. Our results imply that $24 PMPM (2%) in CMS savings would require an incremental standardized bid discount of at least $63 PMPM (7%), the approximate difference in standardized bid discounts between data points in Panels 1 and 2. With current rebate percentages and the 2022 distribution of Star Ratings, CMS thus captures only 38% of incremental savings for MA plans. To minimize participation disincentives and reward larger bid discounts, CMS could consider an increasing marginal rebate bracket approach (eg, 25% share to plans for the first $50 PMPM savings below benchmark, 50% share for the next incremental $50 PMPM savings).

Third, CMS needs strong controls over risk score and star rating growth. If future innovation models expand on VBID and promote new wellness benefits or extend flexibilities for targeted high-value care, CMMI could introduce corridors or caps on risk score growth for participants. These model design features are already used elsewhere in Medicare payment. The ACO REACH model uses a 3% symmetric cap on risk score growth for 2026, and the new LEAD model anticipates using risk score growth caps between 3%-8%.^9,10^ Similarly, CMMI might introduce tighter controls on star rating growth by narrowing or reweighting the measures that might improve among model participants. For example, growth that is driven by quality measures specifically tied to patient outcomes or those related to nutrition and well-being would ensure model participants are financially rewarded only for improvements related to CMS priorities for Medicare beneficiaries.

Beyond testing another VBID-like model, CMMI might use the next MA innovation model as an opportunity to test new payment methodologies for risk adjustment or quality bonuses. For risk adjustment, CMMI could rely less on provider- and plan-recorded diagnoses or implement a budget neutral system, such as the risk transfer mechanism for ACA-compliant individual and small group health plans that fully de-links risk score growth from payments.^11^ CMMI could similarly test a revamp of the star ratings system to modify how star ratings impact plan payments or develop quality measures specific to the innovation model. The 2027 Part C and D proposed rule included a solicitation of comments on these topics.^12^ Several professional organizations and policy groups like the American Medical Association, Arnold Ventures, and Paragon Institute have advocated for a budget-neutral quality bonus program and/or redesigning the 5% benchmark applied to higher-rated plans.^13,14,15^

## Conclusions

Our simulations highlight that the financial sustainability of MA innovation models like VBID is dependent on lower standardized bids in relation to the model parameters for addressing costly risk score and star rating growth effects. Additional tools and model safeguards may be needed to promote savings while preserving participation incentives. In VBID, a 0.6% bid discount was insufficient to fund additional payments driven by 1.5% risk score growth and 5.1% star rating growth. CMS would have required larger bid discounts, lower risk score growth, lower star rating growth, or some combination of those three things to achieve budget neutrality in VBID. As CMS considers future MA innovation models, they might consider implementing minimum standardized bid discounts, a marginal rebate bracket approach, and stopgap measures or alternative methodologies for risk scores and star ratings to ensure model sustainability and promote positive value to Medicare.

## Supplementary Material

qxag199_Supplementary_Data
