# Supplementary material for: The cost of risk score and star rating growth: insights for future Medicare advantage innovation models
Source: Health Aff Sch. 2026 Jul 31;4(8):qxag199. doi: 10.1093/haschl/qxag199 (PMC13455354; doi:10.1093/haschl/qxag199)
Supplement: Supplementary file 1 [file qxag199_supplementary_data.zip › technical_appendix_RR.docx]

**Technical Appendix**

**Background.** Risk scores are factors used to adjust MA plan payments to reflect the predicted costs of enrollees based on their demographics (e.g., age, sex, dual eligibility for Medicaid) and diagnoses (e.g., diabetes, cancer, congestive heart failure).^1^ Star ratings are measured on a 1 to 5 scale in increments of 0.5 and reflect plans’ performance across domains related to care management, member experience, and customer service. Higher rated plans with at least 4.0 stars earn a 5% quality bonus payment from CMS and a larger rebate, or proportion of the difference between the plan bid and benchmark.^1^ Plans with 3.0 stars or lower collect 50% of the difference, plans rated between 3.5 and 4.0 stars collect 65% of the difference, and plans with 4.5 stars or higher collect 70% of the difference.^2^

**Parameters.** In Figure 5.5 of their most recent VBID evaluation report, CMS reports that VBID plan risk scores in 2022 were 1.297 on average and estimates that they would have been 1.278 (i.e., 0.02 points or 1.5% lower) in the absence of VBID.^3^ In Figure 7.1 of the same report, CMS reports that VBID plan star ratings in 2022 would have been 0.19 points lower in the absence of VBID.^3^ Since star ratings can only take discrete values in increments of 0.5, and each value can have a different impact on MA payments (see *Calculated Outcomes* section below), we also considered the distribution of Star Ratings. We translated the 0.19 evaluation estimate to imply that 38% (=0.19/0.5) of plans would have had a star rating 0.5 lower in the absence of VBID, and present these estimates, based on CMS’ 2022 national distribution of star ratings in Table A-1 below.^2^

Table A-1. Distribution of 2022 MA Star Ratings

| Star Rating | CMS Reported % of Beneficiaries | Calculated % of Beneficiaries in absence of VBID |
| --- | --- | --- |
| 5.0 | 26.59% | 16.49% |
| 4.5 | 33.21% | 30.69% |
| 4.0 | 29.87% | 31.14% |
| 3.5 | 8.49% | 16.61% |
| 3.0 | 1.80% | 3.00% |
| 2.5 | 0.03% | 0.21% |
| 2.0 | 0.00% | 0.00% |

Notes: CMS’ original estimates for the percent of beneficiaries in plans with the following star ratings included all MA plans that were offered in 2022.

To simulate star rating growth, we implemented an algorithm that incrementally shifted a share of weight for each star rating category until the target weighted average star rating across plans was achieved. Because we lacked information on the distributional shift in star rating categories associated with overall star rating growth, we assumed iterative 0%, 0.05%, 0.1%, 0.2%, 0.3%, and 0.4% shifts in the weight from plans in 5, 4.5, 4, 3.5, 3, and 2.5-star rating categories, respectively, to the next highest category until target average star ratings, matching the desired star rating growth, were achieved.

In Figure 5.2 of the VBID evaluation report, CMS reports that VBID plan standardized bids in 2022 were $900 and would have been $905 (i.e., $5 higher) in the absence of VBID.^3^ The average benchmark for VBID plans, which is what MA plans bid against, was not reported in the VBID evaluation reports but is determined based on geographic and other adjustments to the fee-for-service (FFS) component of monthly United States Per Capita Costs (USPCC). CMS reported the FFS USPCC for 2022 was $1,028.38.^4^

**Calculated Outcomes.** The calculations of base and rebate payments, which sum to total payments by CMS to VBID plans, apply documented MA payment methodology. The base payment is the standardized bid multiplied by the risk score and coding intensity adjustment (which is 1/1.059). The rebate is the benchmark minus standardized bid, multiplied by the product of the rebate percentage, risk score, and coding intensity adjustment. The rebate percentage is a function of the star rating—50% for star ratings of 3.0 or lower, 65% for star ratings of 3.5 or 4.0, and 70% for star ratings of 4.5 or 5.0. The benchmark is also a function of the star rating; plans with star ratings of 4.0 or higher receive a 5% higher benchmark.

**Sensitivity Analyses**. We conducted several sensitivity analyses to test the robustness of our results by adjusting various input parameters for our simulations, holding all else constant (Table A-2). First, we tested the impact of the standardized bid amount for the base case in the absence of VBID, increasing and decreasing it by 10% (from $905 to $990.50 and $814.50). Because we calculate savings as a percent of the baseline PMPM amount, greater standardized bids would require lower bid discounts from plans for CMS to achieve budget neutrality or 2% savings (excluding any changes to the benchmark). Increasing (and decreasing) the benchmark amounts by 10%, in addition to the standardized bids, did not mechanically impact our results.

We also updated the star ratings distribution using the national 2026 distribution from the star ratings fact sheet and applied the same calculations highlighted in Table A-1.^5^ We tested this to understand how results may have varied if a lower percentage of plans had ratings meeting quality bonus program thresholds, as 2022 star ratings were the highest on average in recent years. Modifying the star rating distribution had minimal impact to our results; we would only expect to see major changes if the proportion of plans receiving quality bonus payments increased significantly (4.0 stars or higher). Similarly, for sensitivity analyses 4-5, adjusting the average overall star rating parameter for VBID plans to values that were 25% higher or lower did not yield any meaningful differences in our results. We also tested changes to the average risk score for the base case, but mechanically these changes did not impact our results.

Finally, in Figure A-1 below, we expanded our grid-search to examine scenarios beyond the 1.5% risk score and 5.1% star rating growth we measured in the main manuscript. For this analysis we labeled: Scenario E representing 2% risk score growth and zero star rating growth, Scenario F representing zero risk score growth and 6% star rating growth, and Scenario G representing 2% risk score growth and 6% star rating growth. These findings strengthen our results in demonstrating that CMS would need to implement greater bid discount amounts to offset increased costs associated with greater risk score and star rating growth.

Table A-2. Alternate Parameters for the Base Case: Minimum Bid Discounts Needed to Achieve Budget Neutrality and 2% Cost Savings (%)

| ***Minimum Bid Discount Needed for Budget Neutrality (%)*** | | | | | | |
| --- | --- | --- | --- | --- | --- | --- |
| Scenario | Main Results: VBID Base Case (Figure 1 of Manuscript) | Sensitivity Analysis 1: Increase mean standardized bid by 10% | Sensitivity Analysis 2: Decrease mean standardized bid by 10% | Sensitivity Analysis 3: Update star ratings distribution using 2026 data | Sensitivity Analysis 4: Increase average overall star rating for VBID plans by 25%, relative to base | Sensitivity Analysis 5: Decrease average overall star rating for VBID plans by 25%, relative to base |
| A | 0 | 0 | 0 | 0 | 0 | 0 |
| B | 2.1 | 1.6 | 2.8 | 2.3 | 2.3 | 2.1 |
| C | 5 | 4.6 | 5.4 | 4.5 | 4.9 | 5 |
| D | 7.3 | 6.4 | 8.4 | 7.1 | 7.4 | 7.3 |
| ***Minimum Bid Discount Needed for 2% Cost Savings (%)*** | | | | | | |
| Scenario | Main Results: VBID Base Case (Figure 1 of Manuscript) | Sensitivity Analysis 1: Increase mean standardized bid by 10% | Sensitivity Analysis 2: Decrease mean standardized bid by 10% | Sensitivity Analysis 3: Update star ratings distribution using 2026 data | Sensitivity Analysis 4: Increase average overall star rating for VBID plans by 25%, relative to base | Sensitivity Analysis 5: Decrease average overall star rating for VBID plans by 25%, relative to base |
| A | 6.7 | 6.3 | 7.2 | 6.1 | 6.7 | 6.8 |
| B | 9.1 | 8.1 | 10.4 | 8.7 | 9.2 | 9.2 |
| C | 11.6 | 10.8 | 12.5 | 10.5 | 11.5 | 11.7 |
| D | 14.2 | 12.8 | 15.8 | 13.4 | 14.2 | 14.3 |

Notes: Scenario A represents cases with zero risk score and star rating growth; Scenario B represents 1.5% risk score growth and zero star rating growth; Scenario C represents zero risk score growth and 5.1% star rating growth; and Scenario D represents 1.5% risk score growth and 5.1% star rating growth.

Figure A-1. Standardized Plan Bid Discounts Needed for CMS Budget Neutrality and Target Savings with Expanded Parameters for Varying Risk Score and Star Ratings Growth

**References**

1. MedPAC. MedPAC Payment Basics: Medicare Advantage Program Payment System [Internet]. 2025 [cited 2026 Mar 26]. Available from: <https://www.medpac.gov/wp-content/uploads/2024/10/MedPAC_Payment_Basics_25_MA_FINAL_SEC.pdf>
2. Centers for Medicare and Medicaid Services. 2022 Star Ratings Fact Sheet [Internet]. 2021 [cited 2026 Mar 26]. Available from: <https://www.cms.gov/files/document/2022-star-ratings-fact-sheet1082021.pdf>
3. Eibner C, Khodyakov D, Taylor EA, et al. Evaluation of the Medicare Advantage Value-Based Insurance Design Model Test: 2020 to 2023 [Internet]. RAND Corporation; 2025. Available from: <https://www.cms.gov/priorities/innovation/data-and-reports/2025/vbid-2020-2023-eval-report>
4. CMS Office of the Actuary. 2022 Rate Announcement: Key Components of non-ESRD USPCC Trends for 2018-2022 [Internet]. [cited 2026 Mar 26];Available from: <https://www.cms.gov/files/document/narrative-supporting-2022-growth-rate.pdf>
5. Centers for Medicare and Medicaid Services. 2026 Star Ratings Fact Sheet [Internet]. 2025 [cited 2026 Jul 21]. Available from: https://www.cms.gov/files/document/2026-star-ratings-fact-sheet.pdf
